# Association Between Nephrolith Size and Location and Grade of Hydronephrosis

**DOI:** 10.3390/life15020321

**Published:** 2025-02-19

**Authors:** Sultan Abdulwadoud Alshoabi, Abdulkhaleq Ayedh Binnuhaid, Abdullatif Mothanna Almohtadi, Halah Fuad Muslem, Abdullgabbar M. Hamid, Fahad H. Alhazmi, Abdulaziz A. Qurashi, Walaa M. Alsharif, Awadia Gareeballah, Amel F. Alzain, Maisa Elzaki, Abdalrahim Tagelsir Elsayed, Salman Althobaiti

**Affiliations:** 1 Department of Diagnostic Radiology, College of Applied Medical Sciences, Taibah University, Al-Madinah Al-Munawwarah 42353, Saudi Arabia; 2 Department of Specialized Surgery, Radiology Section, Faculty of Medicine, Hadhramout University, Al Mukalla, Yemen; 3 Ibb Scan Medical Diagnostic Center, Ibb City, Yemen; 4 Department of Internal Medicine, Dr. Suliman Al Habib Hospital Altakhasosi, Riyadh 12344, Saudi Arabia; 5 Radiology Department, Rush University Medical Group, Chicago, IL 60612, USA; 6 Family and Community Medicine and Medical Education Department, Faculty of Medicine, Taibah University, Al-Madinah Al-Munawwarah 42353, Saudi Arabia

**Keywords:** hydronephrosis grades, pelviureteric junction (PUJ), upper ureter (UU), midureter (MU), lower ureter (LU), vesicoureteral junction (VUJ)

## Abstract

This research investigated the unstudied impact, in 416 cases of stone-induced hydronephrosis detected radiographically in 369 patients, of stone size on the stone’s location in the urinary tract and on the hydronephrosis grade. Most (62.5%) of the hydronephrosis cases were Grade 2; 17.1%, Grade 3; 10.6%, Grade 4; and 9.9%, Grade 1. The mean size of the stones reported in the renal pelvis, pelviureteric junction (PUJ), upper ureter (UU), midureter (MU), lower ureter (LU), and vesicoureteral junction (VUJ) that caused hydronephrosis were 23.03 ± 8.97 mm, 15.56 ± 6.59 mm, 12.91 ± 6.02 mm, 11.05 ± 4.27 mm, 10.41 ± 4.80 mm, and 6.73 ± 3.28 mm, respectively. The mean size of Grade 1-causing stones was 16.63 mm; Grade 2, 11.49 mm; Grade 3, 15.69 mm; and Grade 4, 21.23 mm. The mean stone size significantly decreased from the renal pelvis, through the PUJ, UU, MU, and LU, and down to the VUJ and increased as the hydronephrosis grade increased from Grade 2 to Grade 4. In conclusion, large-size stones were predominantly located in the renal pelvis and PUJ, with few located in the lower ureter, and no large stones reached the VUJ. Small-size stones were mostly located in the VUJ, with only one stone in the PUJ, and no small-size stones were present in the renal pelvis. Large-size stones caused severe hydronephrosis, and small-size stones caused grade 2 hydronephrosis. Increases in stone size decreased its passage rate in the ureter and increased the chance of causing high-grade hydronephrosis. These results alert urologists to adopt faster therapeutic procedures for larger stone sizes to reduce renal damage caused by obstructive uropathy.

## 1. Introduction

Hydronephrosis is defined as the dilatation of the renal collecting system, including the renal pelvis, infundibula, and calices [1]. The Society of Fetal Urology (SFU) grading system of hydronephrosis grades is scored as follows: Grade 0: no dilatation; Grade 1: renal pelvis dilatation; Grade 2: mild caliceal dilatation; Grade 3: severe caliceal dilatation; and Grade 4: parenchymal atrophy [2,3]. The cause of hydronephrosis may be obstructive or non-obstructive, but obstructive causes are more harmful to the kidneys [4]. The most common obstructive cause in adults is renal calculi, which can be found in the following five parts of the kidney and ureter: the renal pelvis, the pelviureteric junction (PUJ), the upper ureter (UU) when cranial to the sacroiliac joint (SIJ), the middle ureter (MU) when overlying the SIJ, the lower ureter (LU) when distal to the SIJ, or the vesicoureteric junction (VUJ) [5,6]. The hydronephrosis grade and the stone location and size have significant roles in patient presentation [7], the selection of treatment methods [8], and outcomes [9].

Medical imaging using ultrasonography, computed tomography (CT), or other imaging modalities is essential in the diagnosis, site determining, size measurement, and management planning of kidney and ureteric calculi. Ultrasonography and CT do not significantly differ in their measurement of the size of urinary calculi [10]. Ultrasonography is a noninvasive, radiation-free imaging method for the detection of ureteric stones [11]. However, the American College of Radiology recommends reduced-dose CT for urinary stone evaluation [12], and the Urological Association of Asia (UAA) guidelines for urinary stone disease indicate that CT is superior to ultrasonography in the detection of ureteric calculi [13].

Stone location and size are critical factors in clinical practice. The stone site in the ureter is the most significant predictor for spontaneous stone passage, and small stones that lie in the lower ureter may be managed conservatively [14,15]. Large stones or those located in the UU or MU and those that cause high-grade hydronephrosis may require more invasive interventions such as percutaneous nephrolithotomy (PCNL) or ureteroscopy [15,16].

The combination of stone size and location is essential in predicting spontaneous stone passage and guiding treatment options. Hydronephrosis is an important medical imaging marker for obstructive uropathy, and hydronephrosis grade is used to assess the severity of renal damage. Understanding the correlation between stone size and location and hydronephrosis grade can provide valuable information for prognostication and management planning. In the literature, no previous study has investigated the impact of stone size on stone location in the urinary tract and the hydronephrosis grade. This study aims to fill this gap by providing more precise insights into the relationship between stone size and location in the urinary tract and stone size and hydronephrosis grade. We hypothesized that small-size stones can pass through the ureter and reach the distal part.

## 2. Methods

### 2.1. Study Population

**Figure 1:**
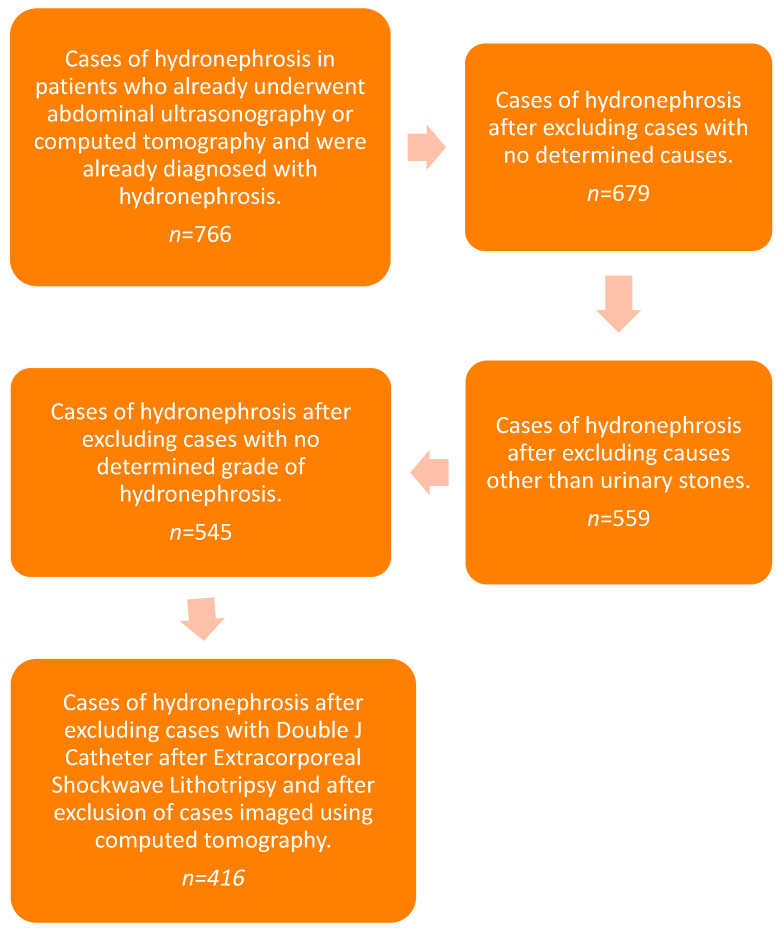
Flowchart of patient population and study design.

This study was a retrospective study that was conducted by investigating the electronic health records of patients radiologically diagnosed with hydronephrosis due to stones in the kidney or the ureter between April 2017 and December 2022 at the Alsafwa Consultative Medical Center in Almukalla, Hadhramout, Yemen. The study involved 416 cases of hydronephrosis detected in 369 patients through renal ultrasonography (*n* = 369, 100%). Patients with no determined causes or other causes than nephroliths, patients with no determined grade of hydronephrosis, patients imaged with CT, and patients with Double J Catheter after Extracorporeal Shockwave Lithotripsy were excluded (Figure 1).

Only cases caused by a stone and with the site and size of the stone determined were included. The patients were aged 1–95 years (mean age: 40.4 ± 16.96 years), and 263 of them (71.3%) were male and 106 (28.7%) were female.

### 2.2. Data Acquisition

**Figure 2:**
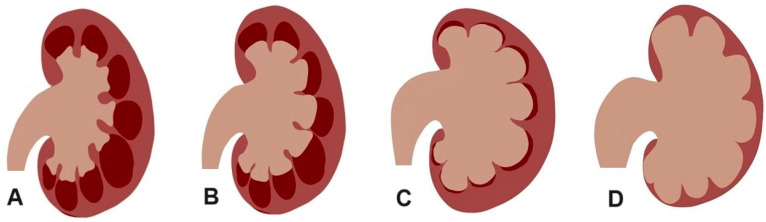
Diagram of the hydronephrosis grades showing (**A**) Grade 1 with renal pelvis dilatation, (**B**) Grade 2 with mild caliceal dilatation, (**C**) Grade 3 with severe caliceal dilatation, and (**D**) Grade 4 with parenchymal atrophy.

**Figure 3:**
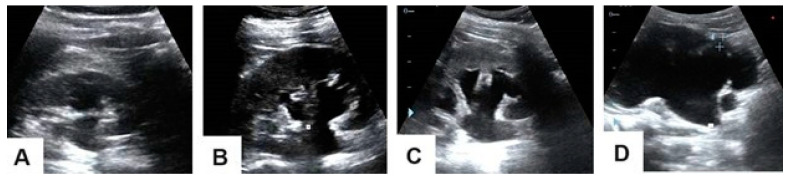
Selected ultrasonography images of hydronephrosis grades showing (**A**) Grade 1 with renal pelvis dilatation, (**B**) Grade 2 with mild caliceal dilatation, (**C**) Grade 3 with severe caliceal dilatation, and (**D**) Grade 4 with parenchymal atrophy.

The hydronephrosis cases were graded (Figure 2) and the sizes of the renal stones that caused them were measured using ultrasonography (Figure 3). The length of the stone was defined as the largest stone diameter that was measured in the axial, coronal, and sagittal sections of ultrasonography [8].

Ultrasonography was performed by a radiologist with 12 years of postdoctoral experience in abdominal ultrasonography using a 3.5 MHz deep curved probe of an (Mindray DC30, Hamburg, Germany) ultrasound machine.

### 2.3. Ethical Approval

This study was approved by the Institutional Review Board (IRB) of the Ethical Research Committee of Hadhramout University’s College of Medicine and Health Sciences in Almukalla City, Hadhramout, Republic of Yemen (Reference No. CM/REC/48/2024), which waived the patients’ informed consent due to the retrospective nature of this study. All the procedures followed the Declaration of Helsinki, as revised in 2013, and all applicable standards and laws.

### 2.4. Statistical Analysis

The data were analyzed using Statistical Package for Social Sciences (SPSS) version 25 (IBM, Armonk, NY, USA). Categorical data are presented as frequency (numbers) and percentage (%), and quantitative data as means ± standard deviations and medians. Shapiro–Wilk, Anderson–Darling, and Kolmogorov–Smirnov^a^ tests were performed and showed non-normality distribution of stone sizes (*p*-value < 0.001); furthermore, histogram analysis was conducted. For the reason of the non-normality distribution of stone sizes, a non-parametric Kruskal–Wallis test was used to compare stone sizes with their locations and with the grades of hydronephrosis. The Kruskal–Wallis 1-way ANOVA test results showed significant differences in stone sizes in different locations (*p*-value < 0.001) and with the grades of hydronephrosis, so the results were further interpreted through Dunn–Bonferroni Post Hoc analysis to compare the groups in pairs to find out which were significantly different. A cross-tabulation test was used to correlate between the location of the stone and the grade of hydronephrosis.

## 3. Results

Unilateral hydronephrosis was predominant (*n* = 369, 88.7%), and only 47 (11.3%) of the cases were bilateral. More than half (224; 53.8%) of the hydronephrosis cases were in the right kidney, and 192 (46.2%) were in the left kidney (Table 1).

The sizes of 35.3% and 39.4% of the stones that caused the hydronephrosis were 5–10 mm and 11–20 mm, respectively, and 28.8%, 21.6%, and 21.2% of the stones were in the LU, renal pelvis, and UU, respectively (Table 2).

The median and mean sizes of the stones significantly varied, decreasing as the stones’ distance from the kidney increased. The Kruskal–Wallis test results with a Chi^2^ of 168.68 and a *p*-value of <0.001 indicate that there is a statistically significant difference in stone size across the six groups of locations (Table 3).

In addition, the stone size significantly varied between locations. The Dunn–Bonferroni Post Hoc tests revealed that the pairwise group comparisons of UU–kidney, UU—VUJ, kidney–PUJ, kidney–LU, kidney–MU, kidney–VUJ, PUJ—LU, PUJ—VUJ, LU—VUJ, and MU—VUJ have an adjusted *p*-value < 0.05, which suggests these groups were significantly different (Table 4).

**Figure 4:**
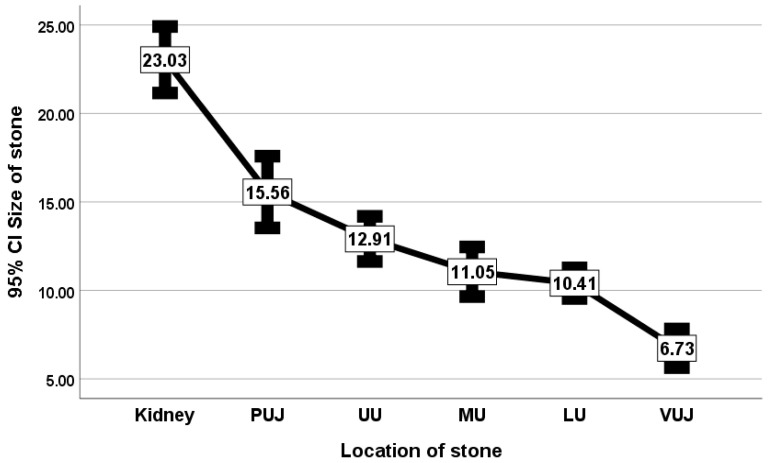
Mean stone size per location of stone with 95% confidence interval.

The mean stone size in the renal pelvis was 23.03 mm; in the PUJ, 15.56 mm; in the UU, 12.91 mm; in the MU, 11.05 mm; in the LU, 10.41 mm; and in the VUJ, 6.73 mm (Figure 4).

The median and mean sizes of the stones also increased as the hydronephrosis grade increased from Grade 2, with Grade 1 stones as outliers; a Kruskal–Wallis one-way ANOVA test demonstrated that there was a significant difference between the grade of hydronephrosis with respect to the stone’s sizes (*p* < 0.001; Table 5).

The Dunn–Bonferroni test revealed that the pairwise group comparisons of Grade 1–Grade 2, Grade 2–Grade 3, and Grade 2–Grade 4 were significantly different in pairs (*p*-value < 0.05) (Table 6).

**Figure 5:**
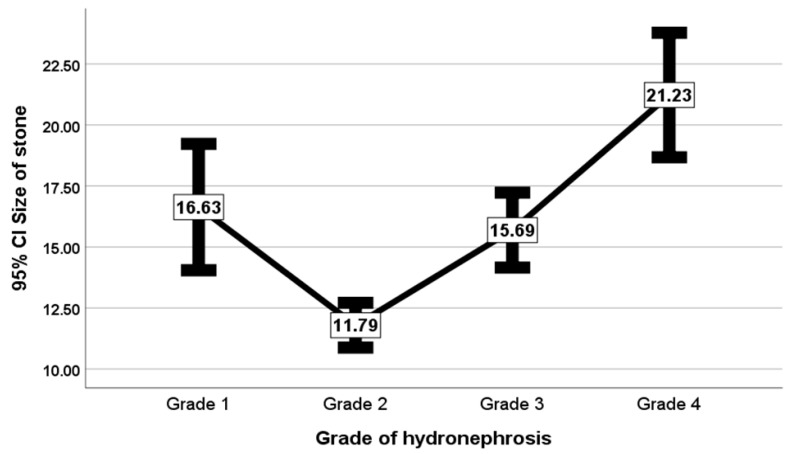
Mean stone size per grade of hydronephrosis with 95% confidence interval.

Specifically, the mean stone size for Grade 1 was 16.63 mm; for Grade 2, 11.79 mm; for Grade 3, 15.69 mm; and for Grade 4, 21.23 mm (Figure 5).

The cross-tabulation test revealed that no small stones were in the renal pelvis, and 50% were in the VUJ. However, 70.7% of stones larger than 20 mm were in the renal pelvis, and none of them reached the VUJ (Table 7).

The cross-tabulation test revealed that 57.7% of stones that caused Grade 3 hydronephrosis were 11–20 mm, and 47.7% and 45.5% of stones that caused Grade 4 hydronephrosis were larger than 20 mm and 11–20 mm, respectively (Table 8).

## 4. Discussion

The location and size of urinary stones that cause hydronephrosis and the hydronephrosis grade are essential factors for hydronephrosis outcomes and management planning. We found that 66.1% of the hydronephrosis cases in this study were mild (Grade 2). Moreover, based on the guidelines of the European Association of Urology that divide stone sizes into <5 mm, 5–10 mm, 11–20 mm, and 20–30 mm [17], the sizes of 35.3% of the stones that caused hydronephrosis in this study were 5–10 mm, and the sizes of 39.4% of the stones were 11–20 mm. These findings align with those of a previous study in which mild hydronephrosis was most common and the sizes of 33.1% and 29.1% of the stones that caused hydronephrosis were 5–10 mm and 11–20 mm, respectively [18]. The current study further found that 28.8% and 8.9% of the stones that caused hydronephrosis were in the LU and the VUJ, respectively. These findings are again comparable with those of a previous study in which 70% of all the ureteric stones were in the distal ureter [19].

In the current study, 23.03 ± 8.97 mm, 15.56 ± 6.59 mm, 12.91 ± 6.02 mm, 11.05 ± 4.27 mm, 10.41 ± 4.80 mm, and 6.73 ± 3.28 mm were the mean sizes of the stones that were reported in the renal pelvis, PUJ, UU, MU, LU, and VUJ, respectively. This result means that large stones are less likely to be able to pass down in the ureter. These findings are consistent with those of Nuss et al., in which the stone size and location influenced stone passage in the ureter, and the passage rate decreased with increasing stone size [20]. The spontaneous passage rates were 87%, 76%, 60%, 48%, and 25% for 1 mm, 2–4 mm, 5–7 mm, 7–9 mm, and >9 mm stones, respectively [21]. Tamsulosin has been found to be capable of improving the passage of 5–10 mm stones [22]. Unfortunately, no similar previous studies are available to compare the mean sizes of stones in different parts of the ureter.

Still, in the current study, a significant variance was observed in the mean sizes of stones that caused different grades of hydronephrosis. The mean sizes of stones increased with increasing hydronephrosis grades by 11.79 ± 7.51 mm, 15.69 ± 6.49 mm, and 21.23 ± 8.39 mm for Grade 2, Grade 3, and Grade 4, respectively. This means that large stones cause higher grades of hydronephrosis. This result aligns with that of a previous study in which cases of no or mild hydronephrosis were less likely than cases of moderate or severe hydronephrosis to have ureteric stones with sizes of >5 mm, with a negative predictive value of 0.876 [23]. In other words, in the absence of hydronephrosis, large ureteric stones (>5 mm in size) are less likely to be present, with an 89% negative predictive value [24]. Iwahashi et al. explained that the hydronephrosis grade worsens as the degree of obstruction increases, as large stones are impacted, which strongly obstruct the ureter [25]. Thus, severe hydronephrosis can be used as an indicator of ureteral stone impaction [13]. In turn, the hydronephrosis grade affects the treatment options and outcomes [26]. Moreover, the presence of ureteral stones with hydronephrosis increases the risk of acute kidney injury in patients with urinary tract infections [27].

Cross-tabulation tests revealed that no stones less than 5 mm were found in the renal pelvis and 50% were in the VUJ. However, 70.7% of stones larger than 20 mm were in the renal pelvis, and none of them reached the VUJ. These results could be explained by the high passage rate of small stones [21]. Our results showed that 57.7% of stones that caused Grade 3 hydronephrosis were 11–20 mm, and that 47.7% and 45.5% of stones that caused Grade 4 hydronephrosis were larger than 20 mm and 11–20 mm, respectively. These results are consistent with those of a previous study in which larger ureteric stones caused higher hydronephrosis grades [28], and with the results of another previous study in which only severe hydronephrosis was associated with a larger stone size [29].

A key outcome of the current study is that it adds new information to the literature regarding the relationship between stone size and location and the relationship between stone size and location and the grade of hydronephrosis. The results revealed reverse relationships between stone size and passage in the ureter. This was explained as small-size stones can easily enter the ureter. There was a linear relationship between an increased stone size and an increased grade of hydronephrosis, with the exception of Grade 1, which may be caused by large-size stones. This can be explained by the fact that large-size stones usually cannot enter the ureter and remain in the renal pelvis with no total ureteric obstruction, which leads to Grade 1 hydronephrosis.

Limitations: Due to this study’s retrospective nature, it was limited to electronic records of the patients, with a low sample size and no follow-up information about the patients.

Future direction: A prospective study with a large sample size, keeping in mind potential confounders such as hydration status, metabolic disorders, and prior treatments, as these factors could influence stone passage and hydronephrosis grade, should be conducted with long-term follow-up to assess whether stone passage predictions align with clinical outcomes.

Conclusion: The combination of stone size and location is essential in predicting spontaneous stone passage and guiding treatment options. Large-size stones were predominantly located in the renal pelvis and PUJ, with a few located in the lower ureter, and no large stones reached the VUJ. Small-size stones were mostly located in the VUJ, with only one stone in the PUJ, and no small-size stones were present in the renal pelvis. Large-size stones caused severe hydronephrosis, and small-size stones caused Grade 2 hydronephrosis. Increased stone size decreases its passage rate in the ureter and increases the chance of causing high-grade hydronephrosis. Understanding the correlation between stone size and location and hydronephrosis grade can provide valuable information for prognostication and management planning in urological clinical practice. This finding should alert urologists to adopt faster therapeutic procedures for larger stone sizes to reduce renal damage caused by obstructive uropathy.

## Figures and Tables

**Table 1:** Characteristics of patients and hydronephrosis.

Variable	Categories	Number (*n* = 464)	Percentage (%)
Sex	Male	263	71.3%
	Female	106	28.7%
	Total	369	100%
Number of hydronephroses per patient	Unilateral	369	88.7%
	Bilateral	47	11.3%
	Total	416	100%
Side of hydronephrosis	Right	224	53.8%
	Left	192	46.2%
	Total	416	100%
Grades of hydronephrosis	Grade 1 (minimal)	41	9.9%
	Grade 2 (mild)	260	62.5%
	Grade 3 (moderate)	71	17.1%
	Grade 4 (severe)	44	10.6%
	Total	416	100%

**Table 2:** Characteristics of stones causing hydronephrosis.

Variable	Categories	Number (*n*) = 549	Percentage (%)
Size of stone	Group 1 (<5 mm)	30	7.2%
	Group 1 (5–10 mm)	147	35.3%
	Group 1 (11–20 mm)	164	39.4%
	Group 1 (>20 mm)	75	18%
	Total	416	100%
Site of stone	Renal pelvis	90	21.6%
	Pelviureteric junction (PUJ)	43	10.3%
	Upper ureter (UU)	88	21.2%
	Middle ureter (MU)	38	9.1%
	Lower ureter (LU)	120	28.8%
	Vesicoureteral junction (VUJ)	37	8.9%
	Total	416	100%

<: less than, >: more than, mm: milliliter, PUJ: pelviureteric junction, UU: upper ureter, MU: middle ureter, LU: lower ureter, VUJ: vesicoureteral junction.

**Table 3:** Comparison of mean sizes of stones in different locations.

	*n*	Median	Mean	Std. Deviation	Mean Rank	Chi^2^	*p*-Value
Kidney	90	23	23.03	8.97	328.68	168.68	<0.001
PUJ	43	15	15.56	6.59	253.77		
UU	88	11	12.91	6.02	205.13		
MU	38	10	11.05	4.27	176.03		
LU	120	9	10.41	4.8	156.71		
VUJ	37	6	6.73	3.28	72.89		
Total	416	12	13.93	8.12			

*n*: number, Std: standard, PUJ: pelviureteric junction, UU: upper ureter, MU: middle ureter, LU: lower ureter, VUJ: vesicoureteral junction.

**Table 4:** Dunn’s Post Hoc test for pairwise comparisons of stone size in different locations.

**Sample 1–Sample 2**	**Test Statistic**	**Std. Error**	**Std. Test Statistic**	***p***	**Adj. *p***
UU–Kidney	−123.55	18	−6.86	<0.001	<0.001
UU—PUJ	−48.64	22.34	−2.18	0.029	0.442
UU—LU	48.42	16.85	2.87	0.004	0.061
UU—MU	29.1	23.31	1.25	0.212	1
UU—VUJ	132.24	23.52	5.62	<0.001	<0.001
Kidney–PUJ	74.92	22.26	3.37	0.001	0.011
Kidney–LU	171.98	16.74	10.27	<0.001	<0.001
Kidney–MU	152.66	23.23	6.57	<0.001	<0.001
Kidney–VUJ	255.79	23.45	10.91	<0.001	<0.001
PUJ—LU	97.06	21.34	4.55	<0.001	<0.001
PUJ—MU	77.74	26.73	2.91	0.004	0.055
PUJ—VUJ	180.88	26.92	6.72	<0.001	<0.001
LU—MU	−19.32	22.35	−0.86	0.387	1
LU—VUJ	83.82	22.58	3.71	<0.001	0.003
MU—VUJ	103.13	27.73	3.72	<0.001	0.003

Significance values have been adjusted by Bonferroni correction for multiple tests. The mean difference is significant at the 0.05 level. PUJ: pelviureteric junction, UU: upper ureter, MU: middle ureter, LU: lower ureter, VUJ: vesicoureteric junction.

**Table 5:** Correlation between stone size and grade of hydronephrosis.

Groups	*n*	Median	Mean ± Std. Dev	Mean Rank	Chi^2^	*p*-Value
Grade 1	41	14	16.63 ± 8.21	256.84	78.55	<0.001
Grade 2	260	9	11.79 ± 7.51	170.13		
Grade 3	71	15	15.69 ± 6.49	255.04		
Grade 4	44	20	21.23 ± 8.39	315.09		
Total	416	12	13.93 ± 8.21			

**Table 6:** Dunn’s Post Hoc test for pairwise comparisons of stone size across different hydronephrosis grades.

Sample 1–Sample 2	Test Statistic	Std. Error	Std. Test Statistic	*p*	Adj. *p*
Grade 1–Grade 2	86.71	20.17	4.3	<0.001	<0.001
Grade 1–Grade 3	1.81	23.55	0.08	0.939	1
Grade 1–Grade 4	−58.25	26.06	−2.24	0.025	0.152
Grade 2–Grade 3	−84.9	16.08	−5.28	<0.001	<0.001
Grade 2–Grade 4	−144.96	19.57	−7.41	<0.001	<0.001
Grade 3–Grade 4	−60.06	23.04	−2.61	0.009	0.055

Significance values were adjusted by Bonferroni correction for multiple tests.

**Table 7:** Cross-tabulation between size and location of stones.

Grade	Renal Pelvis	PUJ	UU	MU	LU	VUJ	Total	*p*-Value
Smaller than 5 mm	0 (0.0%)	1 (3.3%)	3 (10%)	2 (6.7%)	9 (30%)	15 (50%)	30	*p* < 0.001
5–10 mm	5 (3.4%)	6 (4.1%)	34 (23.1%)	19 (12.9%)	65 (44.2%)	18 (12.2%)	147	
11–20 mm	32 (19.5%)	28 (17.1%)	42 (25.6%)	15 (9.1%)	43 (26.2%)	4 (2.4%)	164	
Larger than 20 mm	53 (70.7%)	8 (10.7%)	9 (12%)	2 (2.7%)	3 (4%)	0 (0.0%)	44	
Total	90 (21.6%)	43 (10.3%)	88 (21.2%)	38 (9.1%)	120 (28.8%)	37 (8.9%)	416 (100%)	

mm: millimeter, PUJ: pelviureteric junction, UU: upper ureter, MU: middle ureter, LU: lower ureter, VUJ: vesicoureteral junction, %: percentage.

**Table 8:** Cross-tabulation between grade of hydronephrosis and size of stones.

Stone Size Grade	<5 mm	5–10 mm	11–20 mm	>20 mm	Total	*p*-Value
Grade 1	1 (2.4%)	8 (19.5%)	23 (56.1%)	9 (22%)	41	*p* < 0.001
Grade 2	28 (10.8%)	121 (46.5%)	80 (30.8%)	31 (11.9%)	260	
Grade 3	1 (1.4%)	15 (21.1%)	41 (57.7%)	14 (19.7%)	71	
Grade 4	0 (0.0%)	3 (6.8%)	20 (45.5%)	21 (47.7%)	75	
Total	30 (7.2%)	147 (35.3%)	164 (39.4%)	75 (18%)	416 (100%)	

<: less than, >: larger than, mm: millimeter, %: percentage.

## Data Availability

The data used to produce this article are available from the corresponding author upon reasonable request.

## Abbreviations

PUJ	Pelviureteric junction
UU	Upper ureter
MU	Midureter
LU	Lower ureter
VUJ	Vesicoureteric junction
CT	Computed tomography
NCCT	Noncontrast CT
SIJ	Sacroiliac joint
UAA	Urological Association of Asia
ANOVA	Analysis of variance
PCNL	Percutaneous nephrolithotomy
SPSS	Statistical Package for Social Sciences
